# Low- versus high-dose trimethoprim-sulfamethoxazole for the treatment of *Stenotrophomonas maltophilia* pneumonia

**DOI:** 10.1017/ash.2025.64

**Published:** 2025-04-15

**Authors:** Bradley Taranto, Lynn C. Wardlow, Kelci Coe, Jose A. Bazan, Jessica Leininger

**Affiliations:** 1 Department of Pharmacy, Mount Carmel East Hospital, Columbus, OH, USA; 2 Department of Pharmacy, The Ohio State University Wexner Medical Center, Columbus, OH, USA; 3 Division of Infectious Diseases, Department of Internal Medicine, The Ohio State University Wexner Medical Center, Columbus, OH, USA

## Abstract

**Objective:**

To compare outcomes of patients treated with low-dose (LD) versus high-dose (HD) trimethoprim-sulfamethoxazole (TMP-SMX) for *Stenotrophomonas maltophilia* pneumonia.

**Design:**

Retrospective cohort study.

**Setting:**

Large academic tertiary-care center.

**Patients:**

Hospitalized adult patients who received at least 8 mg/kg/day of TMP-SMX for at least 96 hours for treatment of *S. maltophilia* pneumonia between October 2012 and September 2022. Patients were included if they were diagnosed with pneumonia based on clinical and radiographic findings at the time of initiation of antibiotics.

**Methods:**

The primary outcome was clinical success at the end of therapy among patients treated with LD (8–12 mg/kg/day) versus HD (>12 mg/kg/day) TMP-SMX. Secondary outcomes included microbiological success, all-cause and infection-related inpatient mortality, infection recurrence, development of TMP-SMX resistance, and incidence of acute kidney injury (AKI) and hyperkalemia.

**Results:**

95 patients were included (LD, *n* = 20 versus HD, *n* = 75). There was no difference in the primary outcome of clinical success at the end of therapy between groups (LD 57% versus HD 65%, *P* = 0.53). Secondary outcomes, including inpatient infection-related mortality (*P* = 0.56), AKI (*P* = 0.61), and hyperkalemia (*P* = 0.34) also did not differ significantly between the LD and HD groups.

**Conclusions:**

No differences in clinical success or adverse events were observed in patients with *S. maltophilia* pneumonia treated with either LD or HD TMP-SMX.

## Introduction

*Stenotrophomonas maltophilia,* an aerobic Gram-negative bacillus, represents an increasingly common cause of nosocomial infections, especially in immunocompromised patients. Due to its intrinsic resistance to multiple antibiotics and propensity for biofilm formation, *S. maltophilia* infections pose a unique therapeutic challenge.^1–4^ Pneumonia is the most frequent clinical syndrome observed with *S. maltophilia* infections and is associated with significant morbidity and mortality, particularly when the initiation of effective antibiotic therapy is delayed.^5–8^

Trimethoprim-sulfamethoxazole (TMP-SMX) is considered the drug of choice for *S. maltophilia* infections, either as monotherapy or in combination with other active agents, due to low rates of *in vitro* resistance^9,10^ and decades of clinical experience without explicit evidence of treatment failure.^11–14^ Many references recommend a weight-based dose of 15–20 mg/kg/day of the TMP component in divided doses, a scheme reminiscent of that which is employed for *Pneumocystis jirovicii* pneumonia.^15,16^ However, considering the established risk of dose-dependent toxicities including nausea and vomiting, hyperkalemia, and nephrotoxicity,^17^ the optimal TMP-SMX dose from both an efficacy and safety standpoint remains unknown.

Clinical data investigating the efficacy of TMP-SMX in *S. maltophilia* infections are scarce. Small prospective observational studies and retrospective cohort studies have not provided definitive results on which to base recommendations. These studies boast similar rates of clinical success with TMP-SMX despite wide variability in median daily dose utilized. Moreover, significant heterogeneity in patient immune status and use of combination therapy in the literature limit the ability to draw broad conclusions.^11–14^ As such, more conservative TMP-SMX dosing regimens of 8–12 and 10–15 mg/kg/day have been suggested by infectious diseases (ID) specialists in recently published guidance documents.^18,19^ We designed a single-center, retrospective, cohort study to evaluate the impact of TMP-SMX dosing on outcomes of patients with *S. maltophilia pneumonia*.

## Methods

### Study design, site, and patient selection

This was a retrospective cohort study performed at The Ohio State University Wexner Medical Center, a tertiary academic hospital in Columbus, Ohio, USA. Clinical outcomes of patients treated with TMP-SMX at doses of 8–12 mg/kg/day (low-dose, LD) versus >12 mg/kg/day (high-dose, HD) of the TMP component were compared. Those who experienced an adverse drug event requiring the TMP-SMX dose to be reduced from HD to LD were included in the HD group. Actual body weight was used to determine the weight-based dose, except in the case of obesity (i.e., actual body weight >120% of ideal body weight), in which case adjusted body weight with an adjustment factor of 0.4 was used.

Patients aged 18–89 years with a respiratory culture positive for *S. maltophilia* and who received TMP-SMX at a dose of ≥8 mg/kg/day for ≥96 hours between October 1, 2012, and September 30, 2022, were screened for eligibility. Patients were included if they were diagnosed with pneumonia based on clinical and radiographic findings at the time of initiation of antibiotics. Exclusion criteria included index *S. maltophilia* isolate non-susceptible to TMP-SMX; creatinine clearance <30 mL/min or receiving hemodialysis or peritoneal dialysis at time of TMP-SMX initiation; and receipt of combination therapy targeting *S. maltophilia* for >50% of the treatment course. Protected patient populations, including prisoners and pregnant women, were also excluded. This study was approved by The Ohio State University Office of Responsible Research Practices Institutional Review Board (IRB).

### Study endpoints

The primary outcome was clinical success at the end of therapy, which was defined as resolution or improvement in the clinical features of infection and no further *S. maltophilia*-specific treatment required. This outcome was determined retrospectively by an ID physician assessor. Pneumonia was defined as the presence of radiographic changes (ie, new or progressive infiltrate or consolidation suggestive of bacterial pneumonia) and at least one clinical feature of infection, such as temperature ≥100.4 °F or ≤95.0 °F, white blood cell count >10,000 cells/mm^3^ or <4,500 cells/mm^3^, >15% immature neutrophils regardless of leukocyte count, new onset or acute worsening of pulmonary symptoms or signs (i.e., cough, dyspnea, sputum production), or need for increased suctioning or ventilatory support in terms of fraction of inspired oxygen (FiO_2_) or positive end-expiratory pressure (PEEP).^20^

Secondary outcomes (defined in the Supplementary Table 1). included microbiological success, infection-related inpatient mortality, all-cause inpatient mortality, infection recurrence, development of TMP-SMX resistance, treatment-emergent AKI and/or hyperkalemia, and adverse effects necessitating TMP-SMX dose reduction or discontinuation.

### Statistical analysis

Given the retrospective nature of this study, sample size was driven by eligible patients rather than statistical power. Descriptive statistics, including median with interquartile ranges (IQR) or calculated proportion as a percentage (%), were used to compare the LD and HD groups for the primary univariate analysis. Quantitative variables were analyzed using the student’s t-test, and qualitative variables were analyzed using Pearson’s chi-squared test or Fisher’s exact test, as appropriate. A multivariable logistic regression model was used to estimate the adjusted odds ratios for the relationship between primary outcome of clinical success and low- versus high-dose of TMP-SMX while adjusting for proven confounders. Variables that were moderately associated (*P* < 0.2) with both the exposure (TMP-SMX dose) and outcome (clinical success) at the univariate level were considered in the model as potential confounders. A forward selection method was utilized adding only one variable at a time, with variables that affected the relationship between the exposure and outcome by 15% or greater considered proven confounders and included in the final model. All statistical tests were performed at a significance level of *P* < 0.05 using SAS statistical software (SAS Institute version 9.3, Cary, NC).

## Results

### Study population

**Figure 1.**
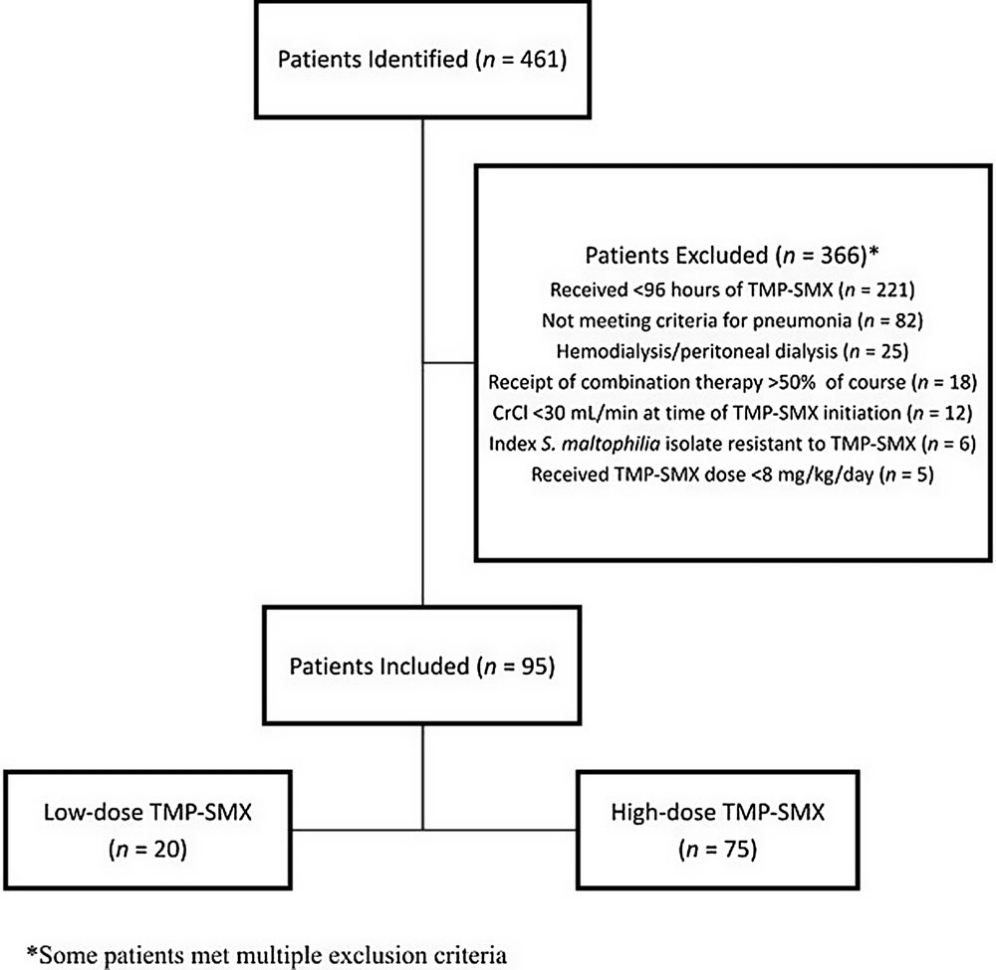
Cohort creation. CrCl, creatinine clearance; TMP-SMX, trimethoprim-sulfamethoxazole.

Of 461 patients with a respiratory culture positive for *S. maltophilia*, 95 were included: 20 in the LD group and 75 in the HD group (Figure 1).

Demographic and baseline characteristics of each group are shown in Table 1. Amongst the total sample, the median age was 61 years, 60 (63%) were male and 25 (26%) were immunocompromised. On the day of index respiratory culture, 71 (75%) were in an ICU, 63 (66%) were mechanically ventilated, and the median SOFA score was 5 (IQR 3-9). The index respiratory culture was polymicrobial (excluding yeast) in 49 (52%) patients and 50 (53%) were managed by an ID consultant. Notably, more patients in the LD group received continuous renal replacement therapy (CRRT) while on TMP-SMX and their baseline serum creatinine at the time of treatment initiation was higher than that of the HD group.

**Table 1.** Baseline characteristics

Characteristic	LD (*n* = 20)	HD (*n* = 75)	*P* value
Age, years	61 [53–73.5]	60 [48–68]	0.12
Male	13 [65]	47 [63]	0.85
Weight, kg	86.5 [69.5–103]	71 [59–88]	0.14
BMI, kg/m^2^	28.3 [22.5–36.5]	25.1 [21–29.3]	0.13
SCr, mg/dL	1.27 [0.75–1.73]	0.67 [0.43–0.92]	<0.0001
CrCl (Cockcroft-Gault), mL/min	58.5 [45–86]	103 [68–162]	<0.0001
CRRT while on TMP-SMX therapy	8 (40)	11 (15)	0.01
SOFA score	8.5 [4.5–11.5]	4 [2–7]	0.01
ICU admission on day of index culture	17 (85)	54 (72)	0.38
Mechanical ventilation on day of index culture	18 (90)	45 (60)	0.01
Duration of mechanical ventilation, days	7.5 [6–15]	11 [7.5–18]	0.38
Immunocompromised (*n* = 25)			0.18
Chemotherapy for cancer	2 (10)	9 (12)	
Solid organ transplant	0 (0)	2 (3)	
High-dose corticosteroids^a^	0 (0)	9 (12)	
Other immunosuppressive medication^b^	0 (0)	3 (4)	
Respiratory culture type			0.69
Sputum/tracheal aspirate	12 (60)	51 (68)	
BAL	3 (15)	16 (21)	
Non-bronchoscopic BAL	5 (25)	8 (11)	
Polymicrobial respiratory culture results	10 (50)	39 (52)	0.87
*Staphylococcus aureus*	3 (15)	6 (8)	0.39
*Pseudomonas* spp.	2 (10)	20 (27)	0.14
*Acinetobacter* spp.	1 (5)	1 (1)	0.38
*Enterobacterales*	4 (20)	18 (24)	1
ESBL-producing gram-negative bacilli	0 (0)	3 (4)	1
Fungi^c^	0 (0)	1 (1)	1
Other^d^	3 (15)	3 (4)	0.11
*S. maltophilia*-positive blood culture	0 (0)	1 (1)	1
Infectious Diseases consultation	12 (60)	38 (51)	0.46

Values presented as median [IQR] or *n* (%). BAL, bronchoalveolar lavage; BMI, body mass index; CrCl, creatinine clearance; CRRT, continuous renal replacement therapy; ESBL, extended spectrum β-lactamase; HD, high-dose; ICU, intensive care unit; LD, low-dose; SCr, serum creatinine; TMP-SMX, trimethoprim-sulfamethoxazole.

a Prednisone dose ≥20 mg/day (or equivalent).

b Includes cyclosporine, azathioprine, and ocrelizumab.

c Includes *Aspergillus* spp.

d Includes *Achromobacter* spp., *Streptococcus* spp., *Enterococcus* spp., and *Corynebacterium* spp.

The median TMP-SMX daily dose received was 10.20 (IQR: 9.6–11.3) mg/kg in the LD group and 14.8 (IQR: 13.7–15.3) mg/kg in the HD group. No difference in median duration of TMP-SMX therapy was observed between groups: 8 (IQR: 6.5–14) days in the LD group versus 10 (IQR: 7–14) days in the HD group. There was also no difference between the LD and HD groups in terms of the number of patients who received combination therapy targeting *S. maltophilia* for part of the treatment course (20% vs 13%, *P* = 0.48), and all such patients received minocycline in combination with TMP-SMX.

## Outcomes

No difference in clinical success at end of therapy was observed between the LD and HD groups (55% vs 63%, *P* = 0.53). Only SOFA score was identified as a proven confounding variable in univariate analysis. Of note, variables that are components of the SOFA score, including mechanical ventilation and those describing baseline renal function (CrCl, SCr, and CRRT), were not considered for the model due to co-linearity. On multivariable regression analysis, there remained no significant difference in clinical success rate after controlling for this variable (aOR 1.63, 95% CI 0.49–5.50). In patients with a repeat respiratory culture collected within 30 days of the end of therapy (*n* = 33), there was no difference in microbiological success between the LD and HD groups. There was also no difference in development of resistance between groups amongst the evaluable population who survived for at least 30 days after the end of therapy (*n* = 63). No differences in other secondary outcomes were noted between groups (Table 2).

**Table 2.** Secondary outcomes

Outcome	Total (*N* = 95) No. (%)	LD (*n* = 20) No. (%)	HD (*n* = 75) No. (%)	*P* value
Microbiological success (*n* = 33)	20/33 (61)	4/6 (67)	16/27 (59)	1
Infection-related inpatient mortality	23 (24)	6 (30)	17 (23)	0.56
All-cause inpatient mortality	28 (29)	7 (35)	21 (28)	0.54
Infection recurrence (*n* = 46)	14/46 (30)	1/10 (10)	13/36 (36)	0.14
Development of resistance (*n* = 63)	5/63 (8)	1/13 (8)	4/50 (8)	1
Acute kidney injury	38 (40)	7 (35)	31 (41)	0.61
Hyperkalemia	17 (18)	5 (25)	12 (16)	0.34
TMP-SMX dose reduction or discontinuation due to AEs	16 (17)	4 (20)	12 (16)	0.74

AE, adverse event; HD, high-dose; LD, low-dose; TMP-SMX, trimethoprim-sulfamethoxazole.

## Discussion

We found no difference in clinical success between LD and HD TMP-SMX for the treatment of *S. maltophilia* pneumonia, even after controlling for the proven confounder of SOFA score. To our knowledge, this is the largest study to date comparing LD and HD TMP-SMX for the treatment of such infections. Infection-related and all-cause inpatient mortality, microbiological outcomes, infection recurrence, and AEs of interest were also similar between groups.

No prospective, randomized trials have compared clinical outcomes in patients treated with different doses of TMP-SMX for *S. maltophilia* pneumonia. To date, only one study has investigated outcomes based on TMP-SMX dose amongst those with such infections.^21^ Patients in this study were stratified into two groups based on dose received after adjusting for renal function: <15 mg/kg/day and ≥15 mg/kg/day. The median doses between groups compared in the previous study were predictably higher than those in our study (12 vs. 16 mg/kg/day and 10.2 vs. 14.8 mg/kg/day, respectively). While this methodological difference makes it difficult to compare findings from this study to ours, the previous study notably also failed to show a difference in clinical success between groups (41% vs. 59%, *P* = 0.24).

In several small, retrospective studies comparing TMP-SMX to other active therapies such as minocycline and fluoroquinolones, no differences were observed in clinical success based on therapy received. Patients included in these studies varied in terms of site of infection and immunocompromised status, and those treated with TMP-SMX received doses consistent with our definition for LD (7.8–10.3 mg/kg/day).^12–14^ In these studies, clinical success was observed in 59–82% of patients treated with TMP-SMX, which slightly exceeds our finding of 55% clinical success in the LD group. This difference may be explained by increased severity of illness in our LD group, as evidenced by the high SOFA score, or due to our strict definition of pneumonia which excluded patients treated for what was likely colonization.

The apparent lack of an obvious relationship between TMP-SMX dose and clinical outcomes as demonstrated in these prior studies, combined with established risks of dose-dependent toxicities associated with TMP-SMX, may form the logical basis for minimizing TMP-SMX dose to avert adverse effects. Our study did not show a difference between the LD and HD groups in terms of AEs of interest, AKI and hyperkalemia, nor in the proportion of patients that discontinued TMP-SMX treatment due to an AE. It should be noted, however, that we did not assess other factors that may influence development of these AEs, including concomitant receipt of medications known to be nephrotoxic or associated with elevations in serum potassium level.

Differences in baseline renal function between groups were present in our study; the median CrCl was 58.5 mL/min in the LD group, compared to 103 mL/min in the HD group. Our institutional antimicrobial guidelines, in keeping with the FDA product labeling, recommend renal dose adjustment of TMP-SMX at CrCl <30 mL/min due to decreased excretion of each component and their metabolites at this level of renal dysfunction.^22^ While TMP elimination has been shown to be strongly correlated with renal function, its impact on SMX elimination is less clear. Inactive metabolites of SMX appear to be more prone to accumulation in renal dysfunction than active unchanged SMX, though the metabolism of SMX has itself been shown to increase as renal function decreases.^23^ As such, the dose-exposure relationship for the combination of TMP and SMX at varying levels of renal dysfunction may be unpredictable and subject to wide interpatient variability.^24,25^ Routine therapeutic drug monitoring of serum TMP and SMX concentrations is not performed at our institution, making it difficult to discern what impact the differences in renal function between groups had on true exposure to each component.

Our study has several important limitations. Given its retrospective nature, it was difficult to account for all variables that may have influenced the TMP-SMX dose chosen. We attempted to control for confounding variables by performing a multivariable logistic regression, though it is possible that we did not account for all variables. Our sample size was also small and included more patients in the HD group than the LD group. Although the sample size is likely reflective of our intentionally stringent definition of pneumonia, it could have impacted our ability to detect differences in outcomes between groups. In addition, a majority of patients had polymicrobial index respiratory cultures, which limited our ability to assess the effect of *S. maltophilia* isolation on clinical outcomes versus other pathogens. Repeat cultures necessary to assess microbiological outcomes and infection recurrence were not always available, and those that may have been collected at outside facilities were not captured. Furthermore, patients with evidence of persistent or recurrent infection may have been more likely to have repeat cultures collected, causing confounding results for infection recurrence and microbiological success by selection bias.

This study found no differences in clinical success, infection-related and all-cause mortality, or renal AEs between LD and HD TMP-SMX, suggesting against the need for higher doses. However, further studies, especially those with larger and more balanced patient populations, are warranted to investigate the true impact of TMP-SMX dose on the outcomes of patients with *S. maltophilia* pneumonia.

## Supporting information

Taranto et al. supplementary material Taranto et al. supplementary material

## Supplementary material

For supplementary material accompanying this paper visit http://doi.org/10.1017/ash.2025.64. click here to view supplementary material
