# Supplementary material for: Low- versus high-dose trimethoprim-sulfamethoxazole for the treatment of Stenotrophomonas maltophilia pneumonia
Source: Antimicrob Steward Healthc Epidemiol. 2025 Apr 15;5(1):e95. doi: 10.1017/ash.2025.64 (PMC12022928; doi:10.1017/ash.2025.64)
Supplement: Taranto et al. supplementary material [file S2732494X25000646sup001.docx]

**Supplemental material**

**Table S1.** Definitions of Primary and Secondary Outcomes

| **Primary outcome** | |
| --- | --- |
| Clinical success | Resolution or improvement in all clinical features of infection (if abnormal at the time of diagnosis) **AND** no further treatment targeted at *S. maltophilia* required – as assessed retrospectively by an ID physician |
| **Secondary outcomes** | |
| Microbiological success | Respiratory culture negative for *S. maltophilia* within 30 days of the end of therapy |
| Infection-related inpatient mortality | Death attributable to *S. maltophilia* infection within 30 days of the index positive respiratory culture or hospital discharge, whichever occurs sooner – as assessed retrospectively by an ID physician |
| All-cause inpatient mortality | Death attributable to any cause within 30 days of the index positive blood culture or hospital discharge, whichever occurs sooner |
| Infection recurrence | Resolution of index infection that is followed by a respiratory culture positive for *S. maltophilia*, with accompanying clinical signs/symptoms of pneumonia, within 30 days of the end of therapy |
| Development of resistance | Repeat respiratory culture positive for *S. maltophilia* exhibiting TMP-SMX non-susceptibility within 30 days of the end of therapy |
| Acute kidney injury (AKI) | Increase in SCr by ≥0.3 mg/dL within 48 hours or an increase in ≥1.5 times baseline within 7 days occurring while receiving TMP-SMX or in the 48 hours following its discontinuation |
| Hyperkalemia | Serum potassium concentration ≥5.5 mmol/L occurring while receiving TMP-SMX or in the 48 hours after its discontinuation |
| Treatment discontinuation or dose reduction secondary to an adverse event | As determined via provider notation in the electronic medical record |
